# Successful Weaning from VA ECMO in a Patient with a Post-Myocardial Infarction Ventricular Septal Defect and a Left Ventricle Apical Aneurysm: A Case Report

**DOI:** 10.3390/healthcare13233006

**Published:** 2025-11-21

**Authors:** Veronica Gagliardi, Laura Tini, Silvia Carbognin, Stefano Angiolini, Giuseppe Gagliardi

**Affiliations:** 1 Department of Anesthesiology and Intensive Care, Ospedale Dell’Angelo, Mestre, 30174 Venezia, Italy; 2 Department of Medicine, University of Padua, 35121 Padova, Italy; 3 Department of Anesthesiology and Intensive Care, Ospedale Santa Maria Della Misericordia, 45100 Rovigo, Italy

**Keywords:** cardiogenic shock, PAC, shunt fraction, transesophageal echocardiography, VA-ECMO

## Abstract

**Introduction:** Although the incidence of mechanical complications of myocardial infarction is decreasing, the associated mortality rate remains high. Such complications require an early diagnosis and multidisciplinary management. In most cases, surgery is the only definitive treatment, despite it being associated with high peri-operative mortality and morbidity. An intra-aortic balloon pump (IABP) or Extracorporeal Membrane Oxygenation (ECMO) may also be required for unstable patients. After the employment of mechanical assistance, ultrasound and chemical parameters are associated with successful weaning, indicating adequate cardiac function, perfusion, and oxygen delivery. **Case presentation:** The aim of this case report is to describe the weaning from the extracorporeal support in a case of post-myocardial-infarction ventricular septal defect (VSD) and Left ventricle (LV) apical aneurysm. The patient underwent surgery for VSD closure and aneurysm exclusion. After the emergency surgery, the patient developed a severe post-cardiotomy cardiogenic shock, which required veno-arterial femoral–femoral extracorporeal membrane oxygenation (VA-ff-ECMO), IABP, and maximal pharmacologic support. During the ICU stay, we weaned the patient from the ECMO support based on transesophageal echocardiography (TEE) imaging and pulmonary artery catheter (PAC) monitoring and quantified the shunt fraction. On the fifth post-operative day, we started the weaning trial. Hemodynamic and ultrasound monitoring showed an adequate cardiac function, and the shunt fraction calculated with both the ultrasound parameters and Fick’s law was acceptable. We removed the ECMO the day after, and the weaning was successful. **Discussion:** Data deriving from the Swan–Ganz catheter has been found to be important in guiding the process of weaning a patient from extracorporeal support. Nevertheless, the TEE played a pivotal role in the decision-making process and in clinical management. We reduced the ECMO blood flow following a real-time echocardiographic cardiac function assessment. **Conclusions:** Following the fundamental guides for both PAC monitoring and TEE imaging, we successfully removed the extracorporeal support, with a positive outcome.

## 1. Introduction

Although the incidence of mechanical complications of myocardial infarction is decreasing, the associated mortality rate remains high. Such complications require early diagnosis and multidisciplinary management [1].

Two example complications are ventricular septal rupture and true aneurysm, and they most commonly occur within the first week after the ischemic event. Cardiogenic shock or acute pulmonary edema are frequent presentations [2]. Regarding the ventricular septal defect, it typically occurs three to five days post-infarction. Echocardiography is required for diagnosis and to determine the size and location of the left-to-right shunt. Anterior and apical ischemic VSDs are caused by anterior infarct, but posterior VSDs are caused by inferior infarcts. In this context, right ventricular infarction or ischemia with severe dysfunction are important features of VSDs caused by acute proximal right coronary occlusion [1]. Ventricular septal rupture results in left-to-right shunt and depends on the relative resistances of systemic and pulmonary circulation, leading to pulmonary over-circulation, reduced cardiac output, systemic hypotension, and organ hypoperfusion [3,4]. In this framework, surgery is the only definitive treatment, even though it is associated with high peri-operative mortality and morbidity. The severity of heart failure is related to the left-to-right shunt [5], and an IABP or ECMO may also be required for unstable patients [6]. Currently, concurrent aneurysm resection is recommended in the presence of a large aneurysm if there is a risk of rupture or large thrombus or if the aneurysm is contributing to recurrent arrhythmias [2,7]. The main issue in the medical management of post-infarct VSD is decreasing the left ventricular afterload while maintaining an adequate systemic pressure [8].

As far as weaning a patient from ECMO is concerned, in the case of concomitant use of an LV venting device, such as an intra-aortic balloon pump (IABP), it is suggested that weaning veno-arterial ECMO (VA-ECMO) is prioritized as it increases the afterload on a failing myocardium with a left-to-right shunt. It is also suggested that the IABP be maintained at 1:1 for unloading and the aortic root be adequately washed.

The ECMO flow rate should be reduced gradually in order to assess the suitability for weaning. It is suggested that flow rates are reduced in increments of 0.5 L/min and then the consequences on MAP and intracardiac pressures are assessed. If the differential pressure is more than 10–15 mmHg, or if significant increases in right-sided filling pressures occur, the patient is not yet ready to be weaned to that level [9]. The extracorporeal Life Support Organization recommends that weaning should be attempted in hemodynamically stable patients on low vasoactive support, employing echocardiography to assess myocardial recovery [10]. The literature has suggested the following parameters associated with successful weaning: aortic VTI = 10 cm; LVEF > 20–25%; lateral mitral annulus peak systolic velocity > 6 cm/s; TAPSE > 10 mm [9,10]. Furthermore, other parameters indicate adequate perfusion and oxygen delivery, such as lactate normalization, SvO_2_ > 65%, recovery from organ failure, the absence of ventricular arrhythmia, optimized fluid balance with a low CVP, and adequate native lung oxygenation capacity with resolution of pulmonary edema and FiO_2_ < 50%. The aim of this case report is to describe the process of weaning a patient from extracorporeal support in a case of post-myocardial-infarction VSD and LV apical aneurysm. The patient underwent surgery for VSD closure and aneurysm exclusion. Our hypothesis is that it is possible to perform effective weaning from extracorporeal support by using data deriving from both echocardiography and PAC (pulmonary artery catheter) monitoring.

## 2. Case Presentation

**Figure 1:**
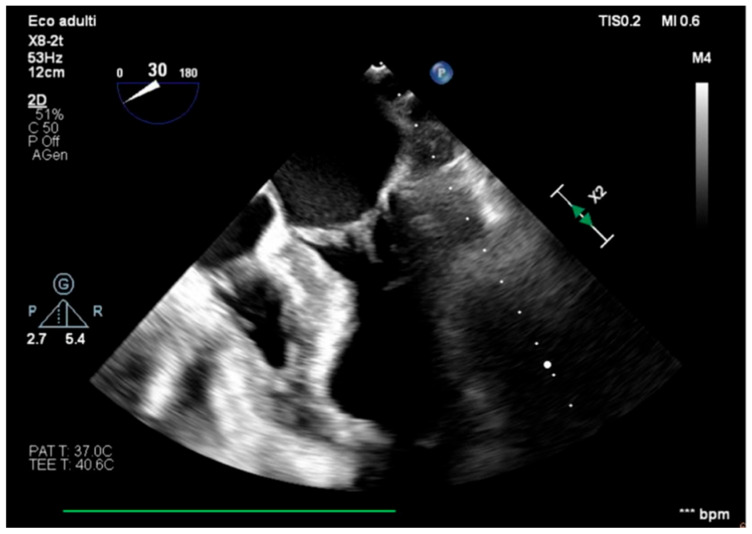
Mid-esophageal four-chamber view.

**Figure 2:**
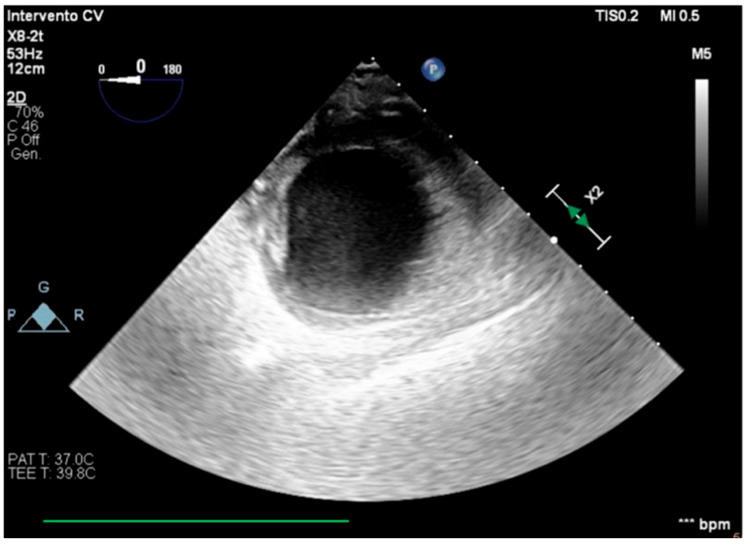
Trans-gastric view, apical level (short axis).

We describe a case of a 75-year-old woman affected by a post-myocardial-infarction ventricular septal defect (VSD) and a left-ventricle (LV) apical aneurysm. The patient underwent surgery for VSD closure and aneurysm exclusion. The patient had a STEMI (ST-elevation myocardial infarction), with evidence of occlusion of the anterior interventricular artery, for which thrombus aspiration and stenting of the left coronary artery and proximal anterior interventricular artery was performed. Then, she developed cardiogenic shock with pulmonary edema and thus required the support of an IABP (intra-aortic balloon pump) of C-PAP and levosimendan in continuous infusion for 24 h. Seven days after the event, a large post-infarct VSD at the apical level with a left–right shunt occurred. She was therefore transported from the spoke center to our hospital and underwent surgical treatment, namely, post-infarct VSD closure and exclusion of a left ventricular aneurysm. The intra-operative transesophageal echocardiography showed concentric LV remodeling, slight dilatation, LVEF 28% (Figure 1), and akinesia of the mid-apical segments in toto with aneurysmal evolution (Figure 2).

She developed a severe post-cardiotomy cardiogenic shock, which required the support of VA-ff-ECMO (veno-arterial femoral–femoral extracorporeal membrane oxygenation) (ECMO flow 100%: 4128 L/min, FiO_2_ 100%) associated with the IABP (1:1, 100%), as well as pharmacologic support with dobutamine 5 mcg/kg/min and epinephrine 0.1 mcg/kg/min as inotropes and norepinephrine 0.1 mcg/kg/min as a vasopressor.

We managed the ECMO support on the basis of TEE (transesophageal echocardiography), PAC monitoring, and arterial (right and left radial) and mixed venous hemogasanalysis, assessing the shunt fraction using both Fick’s law and ultrasound parameters. The timetable of our work is shown in Table 1.

On the first post-operative day (T_0_), the arterial hemogasanalysis and PAC monitoring detected parameters suggesting adequate oxygenation and perfusion. The left radial artery hemogasanalysis showed pH 7.50, pO_2_ 78 mmHg, pCO_2_ 42 mmHg, and lactate 2.5 mmol/L, overlapping with the sample from the right radial artery. From the PAC, we detected PAP 38/20 mmHg, PAPi 1.8, PCWP 28 mmHg, and SvO_2_ 72%.

**Figure 3:**
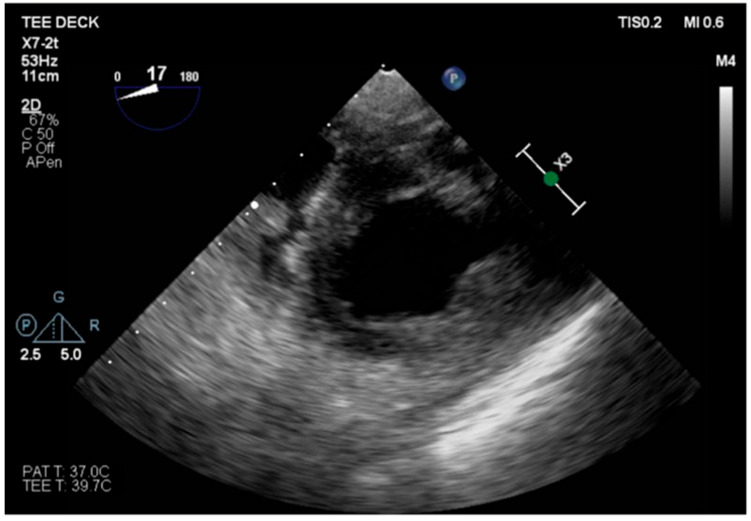
TG mid-papillary level, the day after surgery (T_0_).

**Figure 4:**
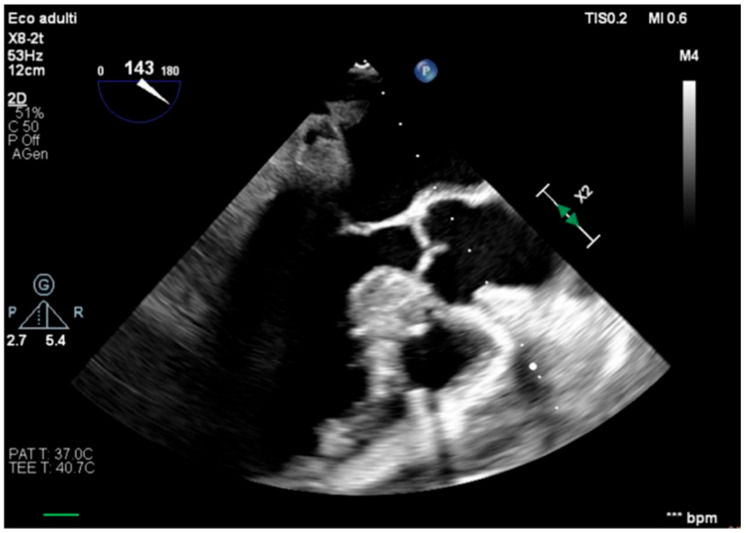
Long-axis view, improved function of the inferior–lateral wall.

**Figure 5:**
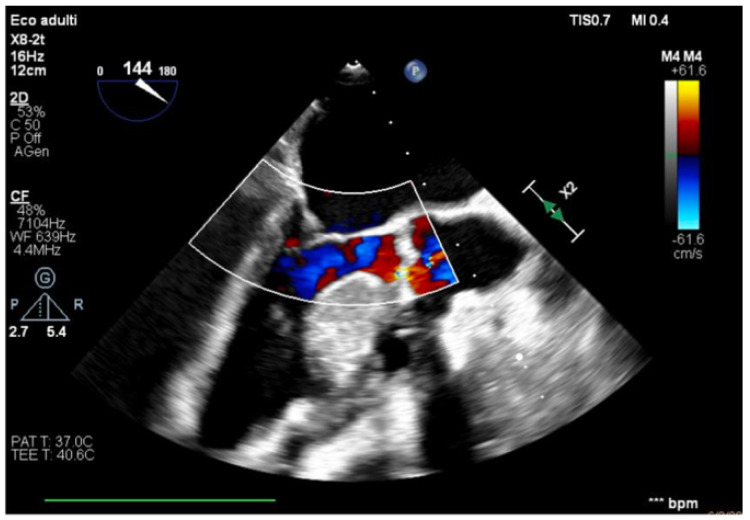
Effective systolic phase, no significant aortic regurgitation.

**Figure 6:**
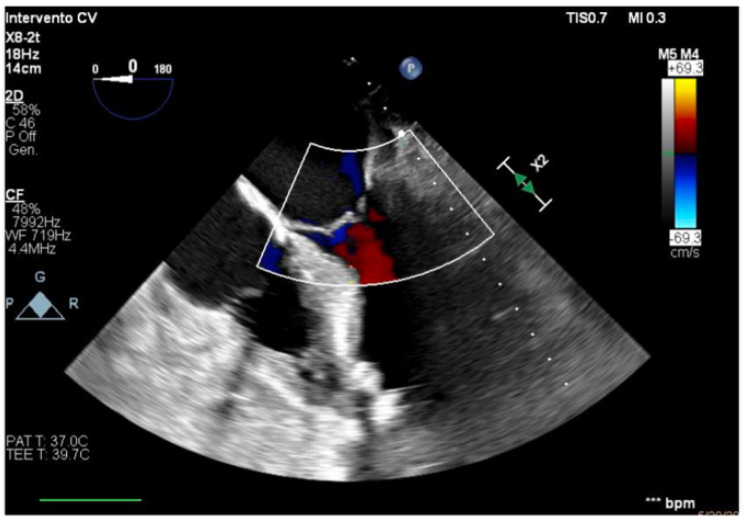
Unloaded right ventricle.

The transesophageal echocardiography showed LVOT-VTI 10 cm; apical, septal, and anterior wall akinesia; preserved function of the medium-basal segments of the inferior and lateral walls (Figure 3, Figure 4 and Figure 5); and RV-FAC 39% with an unloaded right ventricle (Figure 6).

Therefore, the systole was effective, also considering the differential systolic–diastolic pressure of 30–40 mmHg. Taking these data into account, the cardiac function was determined to be recovering. The infusions of epinephrine and norepinephrine have been gradually stopped. Dobutamine has been reduced to 2.5 mcg/kg/min. Moreover, levosimendan 0.12 mcg/kg/min in continuous infusion for 24 h has been administered to improve the weaning success probability and to improve microcirculation recruitment.

IABP was set at 1:2 100% because of the high heart rate for atrial fibrillation (HR 105–120 bpm), for which therapy with digossine and amiodarone has already been introduced. The ECMO flow was reduced to 2.0 L/min (50% of the predicted value) with FiO_2_ 70%, maintaining adequate perfusion and oxygen delivery. After 15 min, the hemogasanalysis showed, from the right radial artery, pO_2_ 68 mmHg and lactate 2.1 mmol/L; and from the left radial artery, pO_2_ 73 mmHg and lactate 2.1 mmol/L.

On the fifth post-operative day (T_1_), we started the weaning trial, reducing the ECMO flow at 1.3 L/min, increasing dobutamine at 5 mcg/kg/min. The invasive monitoring showed SvO_2_ 76%, PCWP 16 mmHg, PAPi 2.2, and lactate 1 mmol/L. At the TEE, we detected the following:

- RV: FAC 40%, RVOT-VTI 16 cm, normal dimensions.
- LV: Improved function of the inferior the lateral walls (LVOT-VTI 15 cm, MAPSE 0.8 mm, e’ lateral 6 cm/s).

Data from the hemodynamic monitoring using the Swan–Ganz catheter and the transesophageal echocardiography in T_0_ and T_1_ are reported in Table 2. Renal and hepatic function were normal.

Moreover, we calculated the pulmonary-to-systemic flow ratio (Qp/Qs) in order to assess the presence and severity of the left-to-right shunt.

Qp/Qs with US parameters:

RVOT VTI (RVOT/2)^2^ = 16 × (2.23/2)^2^ = 19.36 = 0.83
LVOT VTI (LVOT/2)^2^    15 × (2.49/2)^2^  23.25
  Qp/Qs = 0.83

Qp/Qs with Fick’s law:

SO_2*Aorta*_ − SO_2*SuperiorCavaVein*_                =     SaO_2_ − ScvO_2_ = 99.3 − 78.8 = 19.2 = 0.83
SO_2*PulmonaryVein*_ − SO_2*PulmonaryArtery*_        SaO_2_ − SvO_2_     99.3 − 76.2 23.1
  Qp/Qs = 0.83

The TEE imaging two hours after the weaning was the same as previously: low lactate values persisted, SvO_2_ reached a steady state of 68%, PCWP was 17 mmHg, and hemodynamic parameters remained stable. Hence, we successfully decannulated the VA ECMO support the day after.

## 3. Discussion

Data deriving from the Swan–Ganz catheter have proven important in guiding the process of weaning a patient from extracorporeal support. Nevertheless, the TEE played a pivotal role in the process, aiding significantly in decision-making and clinical management. We performed a reduction in the ECMO blood flow using a real-time echocardiographic cardiac function assessment. We reported data detected in T_0_ and T_1_, cardiac function assessments are often repeated more than once a day. We employed transesophageal echocardiography due to our team’s familiarity with this tool and because it provides more precise measurements given the better acoustic window, with high reproducibility compared to transthoracic echocardiography. We did not encounter any complications related to TEE employment. We used transesophageal echocardiography to monitor real-time left ventricle distention and function as it can easily detect LV distention or failure of the aortic valve opening. TEE also provides information about intravascular volume status and preload, identifying patients who might require LV unloading strategies [11]. The method of using ultrasound to calculate the Qp/Qs ratio is a useful tool; it confirmed the shunt fraction calculated with the thermodilution and Fick’s law. We consider this value to be an acceptable shunt fraction, with no impact on the weaning. The residual defect allowed for the weaning of the patient from extracorporeal support.

Swan–Ganz monitoring in VA ECMO is the gold standard by which to measure the pulmonary capillary wedge pressure and the pulmonary systolic and diastolic pressures, providing information on the function of the right and left sections. Moreover, with respect to this kind of patient, it is the only means that allows for the measurement of mixed venous saturation, which is fundamental to detecting low cardiac output syndrome, and it offers systemic resistances to titrate the vasopressors and vasodilators. Regarding PAC-derived parameters, PAPi showed good discrimination for successful outcomes in VA ECMO weaning. Among these patients, higher PAPi during the 24 h preceding a weaning trial was associated with a higher likelihood of success [12]. Nevertheless, this information must be integrated with echocardiographic data, and the patient would be assessed as ready for decannulation if the LVEF was sufficient, i.e., with no greater than moderate right ventricular dysfunction [13].

Within this framework, transesophageal echocardiography integrates this information, providing data about the function of both the left and right ventricles, of the status of the valvular apparatus, and of possible iatrogenic or myocardial infarction-related complications. With these tools, we can assess the effect of any changes in the pharmacological or mechanical supports.

TEE can provide a better acoustic window than TTE, given the limitations of surgical wounds, medications, and drainages, and could prove superior in identifying the mechanical complications of myocardial infarction, such as ventricular septal rupture, papillary muscle rupture leading to acute mitral regurgitation, left ventricular free wall rupture, left ventricular pseudoaneurysm, and true aneurysms.

Ultimately, we expect TEE to provide reliable and accurate data regarding the cardiac function in a context where the Swan–Ganz catheter cannot analyze the cardiac output or other hemodynamic parameters.

As far as the LV venting strategy is concerned, we decided to keep the IABP before surgery. Within this framework, the IABP reduces the LV afterload—an important issue in the management of a post-myocardial infarction VSD—and indirectly reduces the LV preload, as the patient did not demonstrate significant aortic regurgitation. This strategy has been found to be effective.

We decided not to employ other venting strategies, such as Impella CP, as a first-line treatment because it could be dangerous given the ventricular septal defect itself [14], and the LV was effectively unloaded with our first-choice strategy.

In the current literature, there are no significant data showing which mechanical LV unloading strategy is superior [15].

Given that our case study concerns a post-cardiotomy refractory shock, we decided to administer levosimendan, which could prove to enhance cardiac recovery as it increases the probability of weaning success [16]. At the same time, we stopped epinephrine due to its inferior outcome and the microcirculation damage related to its use [17].

## 4. Conclusions

Following the fundamental guides for both PAC monitoring and TEE imaging, we successfully removed extracorporeal support, with a positive outcome. The parameters after weaning could be superimposed on the previous ones, and there were no signs of hypoperfusion. We then progressively weaned the patient from both the inotropic support and the IABP 48 h after the removal of the ECMO. In the future, it might be interesting to assess the Qp/Qs value after the removal of the ECMO and to verify if a shunt fraction is present without confounding factors.

## Figures and Tables

**Table 1:** Timetable.

Day 0	Day 1 (T_0_)	Day 5 (T_1_)	Day 6	Day 7
The patient underwent the surgical correction of the VSD	First assessment with the Swan–Ganz catheter and transesophageal echocardiography	Successful weaning trial	Decannulation of ECMO	Removal of IABP

**Table 2:** Data from Swan–Ganz catheter and the transesophageal echocardiography in T_0_ and T_1_.

Parameter	T_0_	T_1_
PaO_2_ (mmHg)	73	68
PCWP (mmHg)	28	16
SvO_2_ (%)	72	66
PAPi	1.8	2.2
Lactate (mmol/L)	2.5	2.1
LVOT VTI (cm)	10	15
RV FAC (%)	39	40

## Data Availability

The original contributions presented in this study are included in the article. Further inquiries can be directed to the corresponding author.

## Abbreviations

The following abbreviations are used in this manuscript:

VSD	Ventricular septal defect
STEMI	ST-elevation myocardial infarction
IABP	Intra-aortic balloon pump
VA-FF-ECMO	Veno-arterial femoral–femoral extracorporeal membrane oxygenation
paO_2_	Partial pressure of oxygen
PCWP	Pulmonary capillary wedge pressure
SvO_2_	Mixed venous oxygen saturation
PAPi	Pulmonary Artery Pulsatility Index
LVOT VTI	Left Ventricular Outflow Tract Velocity Time Integral
TAPSE	Tricuspid Annular Plane Systolic Excursion
MAPSE	Mitralic annular plane excursion
CVP	Central venous pressure
pCO_2_	Partial pressure of carbon dioxide
PAC	Pulmonary artery catheter
LVEF	Left ventricle ejection fraction
TEE	Transesophageal echocardiography
LV	Left ventricle
RV	Right ventricle
HR	Heart rate
RV FAC	Right ventricle—Fractional Area Change
RVOT VTI	Right Ventricular Outflow Tract Velocity Time Integral
